# Early life factors, blood pressure percentile trajectories, and elevated blood pressure in children

**DOI:** 10.1038/s41390-025-04307-3

**Published:** 2026-04-30

**Authors:** Celeste D. Butts-Jackson, Lea Ghastine, Keia R. Sanderson, Cathrine Hoyo, Chantel L. Martin

**Affiliations:** 1 Department of Epidemiology, Gillings School of Global Public Health, University of North Carolina, Chapel Hill, NC USA; 2 Department of Population Health Management and Policy, North Carolina Agricultural and Technical State University, Greensboro, NC USA; 3 Johns Hopkins Children’s Center, Baltimore, MD USA; 4 Department of Medicine, UNC Kidney Center, Division of Nephrology and Hypertension, University of North Carolina at Chapel Hill, Chapel Hill, NC USA; 5 Department of Biological Sciences, North Carolina State University, Raleigh, NC USA; 6 Center for Human Health and the Environment, North Carolina State University, Raleigh, NC USA; 7 Center for Environmental Health and Susceptibility, University of North Carolina Chapel Hill, Chapel Hill, NC USA

## Abstract

**Background:**

Elevated blood pressure (BP) in childhood is a predictor of adult hypertension.

**Methods:**

We characterized existing blood pressure (BP) percentile trajectories in children aged 3–9 years and examined factors associated with BP trajectory and elevated BP among 1040 participants in a prospective birth cohort study in Durham, North Carolina (2005–2011). We obtained BP measurements from medical records and demographic, social, and behavioral characteristics from questionnaires. We identified systolic blood pressure (SBP) percentile trajectories using group-based trajectory modeling as well as diastolic blood pressure (DBP) percentiles as a secondary outcome. We then calculated associations between trajectory group and covariates of interest using multinomial logistic regression. We also estimated the association of these covariates with elevated BP using generalized estimating equations.

**Results:**

We identified four distinct SBP percentile trajectories (lower stable, lower increasing, higher stable, and higher decreasing) and three distinct DBP percentile trajectories (lower increasing, higher decreasing, and higher stable). Birthing parent race and ethnicity, pre-pregnancy BMI, relationship status, educational attainment, and gestational diabetes status were significantly associated with offspring SBP percentile trajectory group assignment. Offspring sex assigned at birth, birthing parent race and ethnicity, pre-pregnancy BMI, smoking, relationship status, educational attainment, and chronic hypertension status were significantly associated with offspring DBP percentile trajectory group assignment. Infant sex, preterm birth, birthing parent race and ethnicity, pre-pregnancy BMI, and chronic hypertension status were significantly associated with offspring elevated BP.

**Conclusion:**

Our results enhance understanding of pediatric SBP and DBP patterns and assist in identifying factors that contribute to elevated BP in children.

**Impact:**

- Our research contributes to understanding of pediatric BP patterns leveraging percentiles which account for age, sex, and height.
- We identified four distinct SBP and three distinct DBP trajectories in children aged 3–9 years.
- We identified associations of several social, demographic, and clinical characteristics with childhood BP trajectories and elevated BP.
- Elevated blood pressure in childhood is a leading precursor for adverse cardiovascular outcomes in adults. Our research may assist in targeted interventions for high-risk groups.

## Introduction

Hypertension is the leading contributor to cardiovascular disease (CVD)-related mortality in the United States (US), resulting in over 500,000 deaths in 2019.^1^ Nearly half of American adults have hypertension.^1^ Elevated blood pressure (BP) in childhood, defined as systolic or diastolic BP at or above the 90th percentile for a child’s age, sex, and height is a strong predictor of adult hypertension. Recent data indicates it affects at least five to seven percent of US children and the prevalence is increasing.^2–4^ Childhood elevated BP is associated with childhood atherosclerosis and target organ damage (e.g., left ventricular hypertrophy).^5–8^

More recent research has leveraged repeated BP measures throughout childhood to identify clusters of children with similar BP patterns over time using group-based trajectory modeling of systolic BP (SBP) and diastolic BP (DBP) measures.^9–13^ Characterizing age, sex, and height percentile BP patterns using repeated BP measures over time offers advantages over using single BP measures, as an individual BP measure may be inaccurate for various reasons (e.g., illness, anxiety, measurement error). Higher SBP trajectories in childhood have been linked to signs of subclinical CVD in young adulthood, including left ventricular hypertrophy,^10^ greater carotid intima thickness,^10^ and elevated BP.^14^ In fact, SBP is reported to be a strong predictor of adult hypertension and other CVD indicators,^3,15,16^ and isolated elevated DBP in childhood^17^ and adolescence^18^ has also been linked to later adverse CVD outcomes in adulthood. Few studies have examined childhood BP trajectories among a racially diverse cohort of US children.

Social drivers, environmental contaminants, biological factors, and behavioral elements shape childhood cardiovascular health. Various lifestyle factors, including adiposity and high dietary sodium consumption, are associated with elevated BP in childhood.^19,20^ Early life health measures such as low birth weight and intrauterine exposure to birthing parent diabetes mellitus (DM) may also lead to higher childhood BP.^21–23^ Social stressors and environmental contaminants, including lower socioeconomic status (SES), intrauterine exposure to air pollution, and early life adversity are also determinants of elevated BP in childhood and CVD morbidity later in life.^2,24,25^ Biological harm caused by chronic stress due to adverse social environments (i.e. structural racism, poverty, early life adversity) and behavioral factors may contribute to inequities across marginalized racial and ethnic groups.^9,10,26–31^ However, few studies have explored how demographic, social, and clinical factors may influence childhood BP percentile trajectories.

Our research aims to (1) characterize existing SBP and DBP percentile trajectories in a racially diverse cohort of US children from ages 3–9 years, and (2) examine demographic, social, behavioral, and clinical factors associated with trajectory assignment and elevated BP in childhood. This research will contribute to our understanding of factors driving elevated BP in childhood which can have lifelong implications for cardiovascular health.

## Methods

### Study design

Our sample includes offspring in the Newborn Epigenetic Study (NEST), an ongoing prospective birth cohort study with pregnant people recruited in two waves between 2005–2009 (*n* = 2681). NEST did not collect information on gender identity, so we will describe the people who gave birth to offspring in gender neutral terms. Recruitment is described in detail elsewhere.^32,33^ Briefly, the first wave of NEST included 936 English speaking pregnant individuals recruited between 2005 and 2008 from prenatal care clinics operating under Duke University Hospital and Durham Regional Hospital Obstetrics in Durham, North Carolina. The second wave comprised 1745 English and Spanish-speaking birthing parent-child pairs who were recruited from 2009–2011. Eligible parent participants were (1) pregnant, (2) at least 18 years of age, and (3) intending to deliver at Duke University Hospital or Durham Regional Hospital. Parent participants were excluded if they had HIV, were planning to give up custody of the offspring, or intended to move away from the area within 3 years after enrollment. Participants completed self- or interviewer-administered baseline surveys collecting information on demographics, medical history, health behaviors, diet, environmental exposures, and home address during pregnancy. Offspring data were abstracted from medical records spanning all health care encounters taking place at Duke University Medical Center facilities from birth and is currently ongoing.

**Fig. 1:**
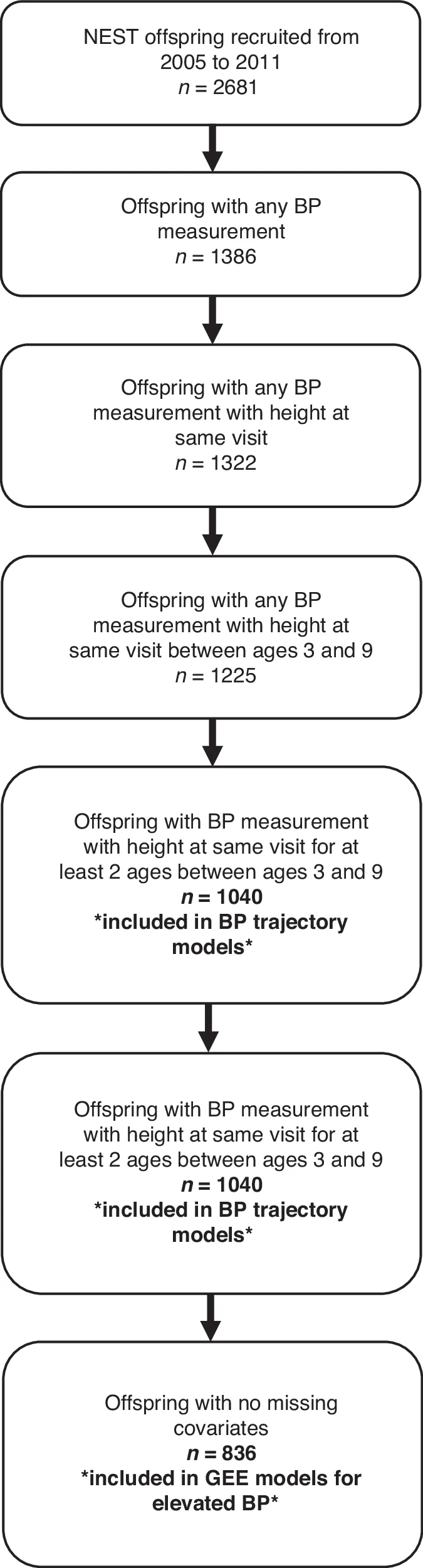
Participant selection flowchart. This diagram illustrates participant selection for trajectory and elevated blood pressure analyses, including eligibility and exclusions based on availibility of data.

The current study includes a subset of NEST cohort offspring who had medical records abstracted from health care encounters with BP and height measurements for at least two separate occasions from ages 3 to 9 years (*n* = 1040). Please see participant selection flowchart (Fig. 1). Children were excluded if they had measures available for fewer than 2 separate years of age (*n* = 1641). Informed consent was obtained from birthing parents at the time of enrollment. This study was approved by the Institutional Review Board at the University of North Carolina at Chapel Hill.

### Blood pressure assessment

Trained study personnel abstracted medical records from inpatient and outpatient health care encounters at Duke University Medical Center facilities to obtain offspring systolic and diastolic BP measurements, age, and height. Age, sex, and height specific systolic and diastolic BP percentiles were attributed to each measure according to American Academy of Pediatrics Clinical Practice Guidelines.^19^ We began with a total of 13,602 BP measures from 1386 NEST offspring. As height is required to calculate BP percentiles, eligible BP measures for this study were required to have a height measurement available for the same encounter. BP measures taken at encounters in which height was not recorded were excluded (*n* = 2389). A substantial portion of children in our study sample had more than one BP measure available for a single year of age. For the purposes of this study, we selected the BP measure closest to the child’s birthday to increase the probability of obtaining the BP taken at the annual well child check. Elevated BP was defined as systolic or diastolic BP at or above the 90th percentile for the child’s age, sex, and height which was abstracted from medical records.

### Variables

Self-administered questionnaires at baseline collected information on birthing parent educational attainment (less than high school, high school graduate/GED, some college, or college graduate), relationship status (never married, married, widowed, living with partner, divorced/separated, or other), race and ethnicity, smoking during pregnancy, chronic hypertension diagnosis, height, and pre-pregnancy weight. Medical record abstractions obtained pregnancy outcomes such as pre-eclampsia, gestational hypertension, offspring sex, birth weight, and gestational length. Birthing parental self-reported race and ethnicity were coded as either Black, White, Hispanic, or Other. Race and ethnicity information was captured differently across the two waves of NEST data collection. The first wave of NEST data collection (2005–2008) coded participant race/ethnicity as either Black, White, Hispanic, or Other, while the second wave of NEST (2009–2011) asked participants to specify their race, ethnicity, and country of birth. Because we are using data from both NEST waves, participant self-identified race and ethnicity are coded in our study as either non-Hispanic Black, non-Hispanic white, Hispanic, or Other. Participants categorized as “Other” were categorized as such due to unknown race and ethnicity or because the number of participants in the sample belonging to that racial or ethnic group was small (e.g., American Indians), *n* = 34. Race and ethnicity were included in this analysis to proxy the different lived experiences and structural exposures of minoritized groups in the U.S.

We dichotomized educational attainment (any college, no college) and relationship status (married or living with partner, neither married nor living with partner). Birthing parental pre-pregnancy body mass index (BMI) was calculated from self-reported height and pre-pregnancy weight and categorized as underweight, normal, overweight, or obese. Parent participant age at delivery was calculated by subtracting the date of birth reported at baseline from the date of delivery abstracted from medical records. We also created a dichotomous hypertensive disorder variable in which a value of 1 indicated that a parent participant reported a chronic hypertension, pregnancy hypertension, or pre-eclampsia diagnosis to maximize the sample size. Offspring preterm birth was defined as delivery at <37 completed gestational weeks, calculated using reported last menstrual period. Small for gestational age (SGA) was determined using newborn sex, birth weight, and gestational age at birth. Newborns were categorized as SGA if they were at or below the 10th percentile for their sex, birth weight, and gestational age in a U.S. reference population of healthy singleton births.^34^

### Statistical analysis

We first examined the distributions of selected demographic, social, behavioral, clinical, birth, and offspring outcomes in our study population using frequencies and means for categorical and continuous variables, respectively. We then used generalized estimating equations (GEE) to estimate the population average effect of participant characteristics on elevated BP status from ages 3 to 9. GEE accounts for correlations between repeated measures within an individual.^35^ The characteristics we examined included demographic (sex, race and ethnicity, participant age at delivery), social and behavioral, (pre-pregnancy BMI, smoking during pregnancy, relationship status, educational attainment), clinical (chronic hypertension, pregnancy hypertension, pre-eclampsia, gestational diabetes mellitus), and birth (preterm birth, SGA) factors. Participants missing any covariates were excluded from this analysis, resulting in a sample size of *n* = 836. Models for potential social and behavioral predictors were adjusted for race/ethnicity and age at delivery, potential clinical predictors were adjusted for social and behavioral characteristics, race/ethnicity, and age at delivery, and potential birth outcome predictors were adjusted for social and behavioral characteristics, clinical characteristics, race/ethnicity, and age at delivery. We adjusted our models in this stepwise approach to capture the upstream confounders associated with each category of variable.

We then conducted group-based trajectory modeling using the SAS function PROC TRAJ, which groups individuals with similar patterns of an outcome,^36^ in this case SBP and DBP percentile, across the study period. We employed PROC TRAJ to estimate predicted values and the shape of the trajectory (i.e., linear, quadratic, cubic) for each group, in this case it predicted trajectories for the available BP measurements between ages 3–9. The procedure estimates the percentage of participants assigned to each trajectory group and their individual posterior probabilities for assignment in that group; participants are assigned to the group for which they have the highest posterior probability, which are the probabilities that children in a given trajectory fit a distinct profile based on BP measures over time.^37^ After constructing a series of models, the final trajectory model was selected based on several considerations; the first was a comparison of Bayesian Information Criterions (BICs), where a lower value indicates a better model fit,^38^ after establishing a maximum number of groups^39,40^ based on previous literature.^10,11,29,30,41^ This also helped to facilitate comparisons across studies. We then considered the average posterior probability (APP) for each group, which is an additional marker of model adequacy; an APP value > 0.70 is recommended.^42^ Additionally, we assessed the number of groups that allowed us to observe variation in trajectories through empirical observation.

We used multinomial logistic regression to compute odds ratios (ORs) and 95% confidence intervals (CIs) estimating unadjusted associations between trajectory assignment and the same demographic, social, and behavioral, clinical, and birth characteristics examined in the elevated BP analysis.

Statistical analyses were completed using SAS 9.4 (SAS Institute Inc., Cary, NC) and R 4.0.2 (R Foundation for Statistical Computing, Vienna, Austria).

## Results

### Demographic, social, behavioral, and clinical factors

The distributions of demographic, social, behavioral, and clinical variables in our study population are displayed in Table 1. The mean age at delivery for birthing parents was 28.4 years with ages ranging from 18–49. Half of our participants (50.7%) identified as Black, 27.7% as White, and smaller proportions (21.7%) identified as Hispanic or other. Approximately 38% of participants had pre-pregnancy BMIs categorized as normal, while a larger proportion (58.1%) had BMIs categorized as overweight or obese. Additionally, most participants (75.3%) reported not smoking during pregnancy, slightly more than half (51.4%) attended college, and 61.1% reported being married or living with a partner. Only 5.5% and 6.5% of our participants reported pre-eclampsia or gestational diabetes diagnoses, respectively. Eight percent and 5.7% reported chronic hypertension and pregnancy induced hypertension, respectively. About half of the infants were assigned male sex at birth (52.3%), there were 141 preterm births which constituted close to 14% of our sample, and 121 (12.2%) infants were SGA. The median number of offspring BP measurements was 4 with a maximum of 7. In Table 2, we describe the proportion of individual measures of BP from ages 3 to 9, characterized as elevated (yes) or non-elevated (no). Overall, the proportion of elevated BP measures was under 14% across the age ranges, with the highest proportion at age 3 (13.4%) and the lowest at age 5 (8.2%).

**Table 1:** Demographic and clinical characteristics of NEST birthing parent-child pairs with offspring BP measurements from ages 3–9 (*n* = 1040).

Characteristics	*N* (%) or Mean (SD)	Missing (*n*)
Birthing parent age at delivery	28.4 (6.05)	0
Birthing parent race/ethnicity		0
Black	527 (50.7%)	
White	288 (27.7%)	
Hispanic	191 (18.4%)	
Other	34 (3.3%)	
Pre-pregnancy BMI		54
Underweight	39 (4.0%)	
Normal	374 (37.9%)	
Obese	333 (33.8%)	
Overweight	240 (24.3%)	
Birthing parent smoking during pregnancy		42
No	751 (75.3%)	
Yes	247 (24.7%)	
Attended college		37
Yes	516 (51.4%)	
No	487 (48.6%)	
Married or cohabitating		44
Yes	609 (61.1%)	
No	387 (38.9%)	
Pre-eclampsia		14
No	969 (94.4%)	
Yes	57 (5.6%)	
Gestational diabetes		44
No	928 (93.2%)	
Yes	68 (6.8%)	
Chronic hypertension		15
No	943 (92%)	
Yes	82 (8%)	
Pregnancy hypertension		19
No	963 (94.3%)	
Yes	58 (5.7%)	
Any hypertensive disorder: (chronic hypertension, pregnancy hypertension, or pre-eclampsia)		20
No	853 (83.6%)	
Yes	167 (16.4%)	
Sex of baby		57
Male	514 (52.3%)	
Female	469 (47.7%)	
Preterm birth		12
No	887 (86.3%)	
Yes	141 (13.7%)	
Small for gestational age		47
No	872 (87.8%)	
Yes	121 (12.2%)	
No. years with BP measurements	3.96 (1.46)

**Table 2:** Proportion of offspring with elevated BP by age in NEST.

	Elevated BP	
	No	Yes
Age 3 (*N* = 714)	618 (86.6%)	96 (13.4%)
Age 4 (*N* = 856)	757 (88.4%)	99 (11.6%)
Age 5 (*N* = 886)	813 (91.8%)	73 (8.2%)
Age 6 (*N* = 640)	587 (91.7%)	53 (8.3%)
Age 7 (*N* = 499)	441 (88.4%)	58 (11.6%)
Age 8 (*N* = 332)	295 (88.9%)	37 (11.1%)
Age 9 (*N* = 191)	173 (90.6%)	18 (9.4%)

### Associations between demographic, social, behavioral, and clinical factors, and elevated blood pressure

Table 3 displays the results of our models exploring associations of demographic, social, behavioral, and clinical factors with elevated BP over ages 3 to 9 years. We found that infants assigned female sex at birth were less likely to have elevated BP (OR = 0.63, 95% CI: 0.50–0.79) compared to infants assigned as male. Infants born to birthing parents who self-identified as Black (OR = 1.78, 95% CI: 1.34–2.36) and Hispanic (OR = 1.82, 95% CI: 1.27–2.59) had higher odds of elevated BP. Infants of birthing parents with pre-pregnancy BMIs categorized as obese and chronic hypertension were also at higher risk of elevated BP with ORs of (OR = 1.56, 95% CI: 1.18–2.05) and (OR = 1.43, 95% CI: 1.00–2.05), respectively. Preterm infants, likewise, had higher odds of elevated BP (OR = 1.49, 95% CI: 1.05–2.10). We did not detect statistically significant associations between elevated BP status and birthing parent age, smoking during pregnancy, relationship status, educational attainment, pregnancy induced hypertension, pre-eclampsia, gestational diabetes, the composite hypertensive disorder category, or small for gestational age. The estimates from sensitivity models controlling for season of birth were similar (Supplementary Table S1).

**Table 3:** Adjusted associations of demographic, social, behavioral, clinical, and birth outcomes with offspring elevated BP (complete cases, *n* = 836).

Characteristics		OR (95% CI)
Demographic characteristics, unadjusted		
Female		0.63 (0.50–0.79)
Birthing parent race/ethnicity		
	White	Ref
	Black	1.78 (1.34–2.36)
	Hispanic	1.82 (1.27–2.59)
	Other	1.06 (0.50–2.27)
Birthing parent age at delivery (years)		
	<35	Ref
	≥35	1.01 (0.76–1.35)
Birthing parent social and behavioral characteristics adjusted for age and race/ethnicity^a^		
Pre-pregnancy BMI		
	Underweight	1.07 (0.59–1.97)
	Normal	Ref
	Overweight	1.20 (0.87–1.64)
	Obese	1.56 (1.18–2.05)
Birthing parent smoking during pregnancy		1.26 (0.96–1.65)
Relationship status		
	Married or cohabiting	Ref
	Not married or cohabiting	1.22 (0.94–1.57)
Educational attainment		
	Attended college	Ref
	Did not attend college	1.11 (0.87–1.43)
Birthing parent clinical characteristics adjusted for age, race/ethnicity, and social and behavioral characteristics^b^		
Chronic hypertension		1.43 (1.00–2.05)
Pregnancy hypertension		0.75 (0.45–1.25)
Pre-eclampsia		1.25 (0.83–1.90)
Any hypertensive disorder: (chronic hypertension, pregnancy hypertension, or pre-eclampsia)		1.23 (0.93–1.64)
Gestational DM		1.16 (0.75–1.79)
Birth outcomes adjusted for age, race/ethnicity, and social, behavioral, and birthing parent clinical characteristics^c^		
Preterm birth		1.49 (1.05–2.10)
Small for gestational age		0.96 (0.67–1.38)

^a^Adjusted for age and race/ethnicity.

^b^Adjusted for age, race/ethnicity, pre-pregnancy BMI, birthing parent smoking during pregnancy, relationship status, and educational attainment.

^c^Adjusted for age, race/ethnicity, pre-pregnancy BMI, birthing parent smoking during pregnancy, relationship status, educational attainment, chronic hypertension, pregnancy hypertension, pre-eclampsia, and gestational DM.

### Systolic blood pressure trajectory analysis and predictors of trajectory

**Fig. 2:**
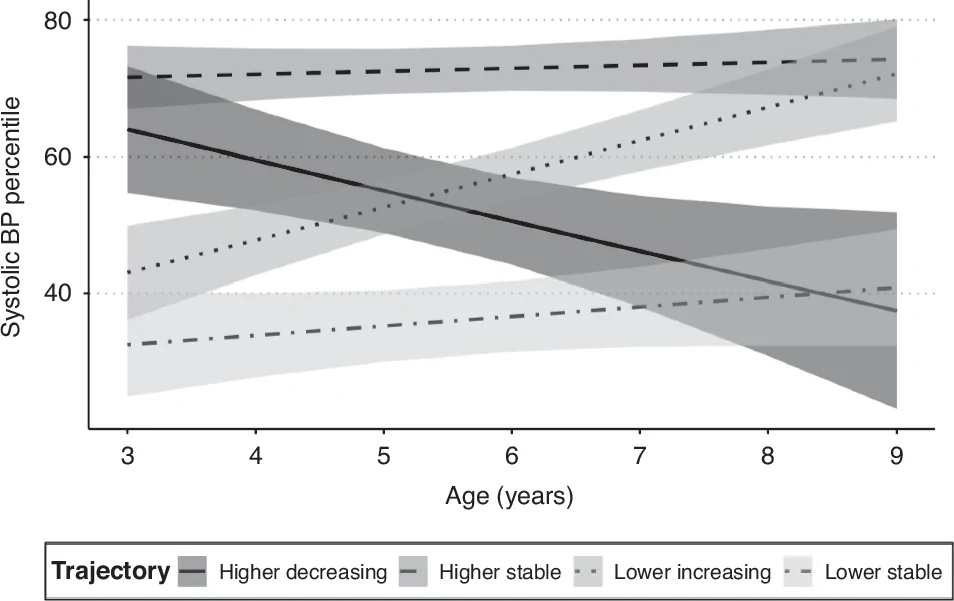
NEST offspring predicted SBP percentile trajectories and 95% CIs, ages 3–9 (*n* = 1040). Lines represent model-estimated mean trajectories for each group. Shaded areas indicate 95% confidence intervals around the estimates.

As displayed in Supplementary Table S2, the four-class SBP trajectory model with all linear shapes yielded the best fit (BIC = −19,270.7, log likelihood = −19,229.02). We also determined that the four-trajectory model best displayed the variation in trajectories whereas other options ‘masked’ groups that fell in between the higher and lower SBP percentile groups. The four distinct SBP percentile trajectory groups, and corresponding percentages of participants in each group determined by APPs were lower stable (*n* = 174, 16.7%), lower increasing (*n* = 434, 41.7%), higher stable (*n* = 313, 30.1%), and higher decreasing (*n* = 119, 11.4%) for children in our study. Trajectories and 95% CIs are displayed in Fig. 2. The APPs, (the group-specific averages of the probabilities of group members belonging to the correct trajectory based on their BP profiles), for the lower stable, lower increasing, higher stable, and higher decreasing trajectory groups were 0.59, 0.70, 0.68, and 0.55, respectively. We want to qualify that our use of ‘stable’ in this analysis refers to consistency in percentile classification over the study period. For a frame of reference, the lower stable group started with lower SBP percentiles which remained lower over time. We designated this group as the referent trajectory. The lower increasing trajectory group started with lower percentiles which increased over the study period. The higher stable trajectory started in the highest percentile and remained high over the study period. The higher decreasing group started with high SBP percentiles which decreased over time. The median (IQR) values for each trajectory group at each age are displayed in Supplementary Table S3.

The results from our multinomial regression models exploring predictors of offspring SBP trajectory group are described in Table 4. In the evaluation of social and economic variables, children of Black birthing parents had higher odds of lower increasing SBP trajectory group (odds ratio (OR) = 1.61, 95% CI: 1.10–2.36), over three-fold higher odds of higher stable SBP trajectory group (OR = 3.76, 95% CI: 2.40–5.89), and higher odds of higher decreasing trajectory group (OR = 2.10, 95% CI: 1.22–3.61) in comparison to offspring of white birthing parents. Children with Hispanic birthing parents similarly had higher odds of lower increasing SBP trajectory group (OR = 3.64, 95% CI: 1.86–7.11), higher odds of higher stable SBP trajectory group (OR = 10.43, 95% CI: 5.14–21.14), and higher odds of higher decreasing trajectory group (OR = 5.53, 95% CI: 2.46–12.44) compared to those with white birthing parents. Additionally, a birthing parent being unmarried and not living with a partner was associated with higher odds of higher stable SBP trajectory group compared to those with birthing parents who were married or living with partner and having a birthing parent that did not attend college was associated with higher odds of lower increasing SBP trajectory group (OR = 1.60, 95% CI: 1.10–2.32), higher stable SBP trajectory group (OR = 3.46, 95% CI: 2.32–5.14), and higher decreasing trajectory group (OR = 2.18, 95% CI: 1.34–3.53) compared to children with birthing parents who attended college. A birthing parental pre-pregnancy BMI that was overweight (OR = 1.74, 95% CI: 1.06–2.87) or obese (OR = 2.08, 95% CI: 1.31–3.30) was associated with increased odds of offspring being in the higher stable SBP trajectory group.

**Table 4:** Unadjusted multinomial logistic regression for predictors of systolic blood pressure (SBP) trajectory membership among NEST (*n* = 1040) using lower stable^a^ as the comparison group.

	Lower increasing	Higher stable	Higher decreasing	*N* missing
	*n* = 434	*n* = 313	*n* = 119	
	OR (95% CI)	OR (95% CI)	OR (95% CI)	
Female	1.23 (0.86–1.77)	1.06 (0.72–1.55)	1.11 (0.86–1.77)	57
Birthing parent race/ethnicity				0
White^b^	Ref	Ref	Ref	
Black	**1.61 (1.10–2.36)**	**3.76 (2.40–5.89**)	**2.10 (1.22–3.61)**	
Hispanic	**3.64 (1.86–7.11)**	**10.43 (5.14–21.14)**	**5.53 (2.46–12.44)**	
Other	0.70 (0.28–1.77)	1.60 (0.60–4.32)	1.48 (0.46–4.77)	
Birthing parent age at delivery (years)				0
<35	Ref	Ref	Ref	
≥35	0.83 (0.54–1.29)	0.66 (0.41–1.06)	0.86 (0.48–1.53)	
Pre-pregnancy BMI				54
Underweight	1.07 (0.43–2.68)	1.05 (0.37–2.96)	1.10 (0.33–3.68)	
Normal	Ref	Ref	Ref	
Overweight	1.04 (0.65–1.66)	**1.74 (1.06–2.87)**	1.33 (0.73–2.44)	
Obese	1.25 (0.81–1.93)	**2.08 (1.31–3.30)**	1.07 (0.60–1.93)	
Birthing parent smoking during pregnancy	0.90 (0.59–1.37)	1.19 (0.78–1.84)	0.91 (0.53–1.59)	42
Relationship status				44
Married or living with partner	Ref	Ref	Ref	
Not married or living with partner	1.23 (0.84–1.79)	**1.80 (1.22–2.68)**	1.28 (0.78–2.10)	
Educational attainment				37
Attended college	Ref	Ref	Ref	
Did not attend college	**1.60 (1.10–2.32)**	**3.46 (2.32–5.14)**	**2.18 (1.34–3.53)**	
Chronic hypertension	**2.35 (1.03–5.36)**	2.23 (0.95–5.25)	1.97 (0.71–5.45)	15
Pregnancy hypertension	0.73 (0.35–1.50)	0.77 (0.36–1.65)	0.85 (0.32–2.23)	19
Pre-eclampsia	1.86 (0.75–4.58)	1.70 (0.66–4.36)	1.47 (0.46–4.68)	14
Any hypertensive disorder: (chronic HTN, pregnancy HTN, or pre-eclampsia)	1.39 (0.83–2.32)	1.34 (0.78–2.30)	1.59 (0.83–3.02)	20
Gestational diabetes	0.97 (0.42–2.27)	2.06 (0.92–4.62)	**2.62 (1.05–6.53)**	44
Birth outcomes				
Preterm birth	1.28 (0.73–2.26)	1.72 (0.97–3.05)	1.26 (0.61–2.61)	12
Small for gestational age	1.20 (0.67–2.15)	1.33 (0.73–2.43)	1.22 (0.57–2.58)	47

*htn* hypertension.

^a^*n* = 174.

^b^Chosen reference as we consider this group “unexposed” to harmful effects of structural racism.

Bold indicates estimates with 95% confidence intervals that exclude the null.

Birthing parent obstetric factors associated with trajectory group for children in our sample included gestational diabetes which was associated with higher odds of higher decreasing trajectory group (OR = 2.62, 95% CI: 1.05–6.53) and chronic hypertension which was associated with a higher odds of lower increasing trajectory group (OR = 2.35, 95% CI: 1.03–5.36). Assigned infant sex, birthing parental age, smoking, gestational hypertension, pre-eclampsia, any hypertensive disorder, preterm birth, and SGA were not associated with SBP trajectory in our population. Sensitivity models controlling for season of birth yielded similar results (Supplementary Table S4).

### Diastolic blood pressure trajectory analysis and predictors of trajectory

**Fig. 3:**
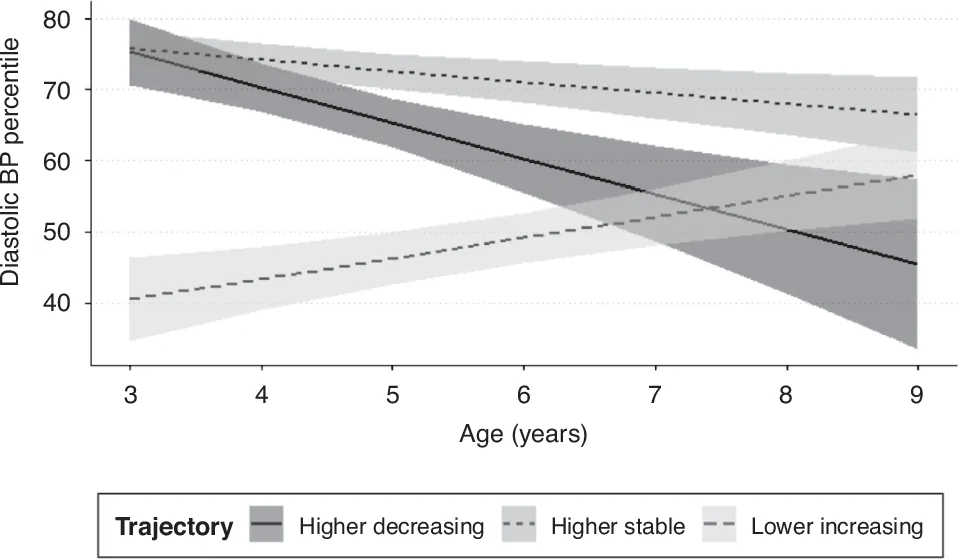
NEST offspring predicted DBP percentile trajectories and 95% CIs, ages 3–9 (*n* = 1040).

The three-class DBP trajectory model with all linear shapes was chosen based on fit (BIC = −18,653.04, log likelihood = −18,621.78), as described in Supplementary Table S5, and observation of the trajectory groups which indicated that empirically three trajectories would allow us to capture more variation than the two-group model. The three distinct DBP percentile trajectory groups, and corresponding percentages of participants in each group were lower increasing (*n* = 166, 16.0%), higher stable (*n* = 717, 68.9%), and higher decreasing (*n* = 157, 15.1%) for children in our study. Trajectories and 95% CIs are displayed in Fig. 3. The APPs, (the group-specific averages of the probabilities of group members belonging to the correct trajectory based on their BP profiles), for the lower increasing, higher stable, and higher decreasing trajectory groups were 0.72, 0.69, and 0.62, respectively. We used the higher stable group as the referent trajectory in this analysis. The median (IQR) values for each trajectory group at each age are displayed in Supplementary Table S6.

The results from our multinomial regression models exploring predictors of offspring DBP trajectory group are described in Table 5. For purposes of brevity, please see table for all odd ratios with 95% confidence intervals. Infants assigned female sex at birth had higher odds of lower increasing trajectory group (odds ratio (OR) = 1.93, 95% CI: 1.35–2.36), an association that we did detect for SBP trajectory. Children of Black birthing parents had lower odds of lower increasing DBP trajectory group and of higher decreasing DBP trajectory group and those with Hispanic birthing parents similarly had lower odds of lower increasing DBP trajectory group and of higher decreasing trajectory group compared to those with white birthing parents. Additionally, a birthing parent smoking during pregnancy was associated with lower odds of higher decreasing group trajectory for offspring (OR = 0.58, 95% CI: 0.37–0.92), whereas we did not find a significant association for SBP, and having a birthing parent that was unmarried or not living with a partner was associated with lower odds of lower increasing and higher decreasing trajectory group compared to those with birthing parents who were married or living with partner. Children of parents that did not attend college was associated with lower odds of lower increasing DBP trajectory group and higher decreasing trajectory group compared to children with birthing parents who did not attend college. A birthing parental pre-pregnancy BMI categorized as overweight was associated with decreased odds of offspring being in the lower increasing DBP trajectory group and a BMI categorized as obese was associated with lower odds of higher decreasing trajectory for offspring. Having a birthing parent with chronic hypertension was associated with lower odds of lower increasing trajectory group in offspring, while having a birthing parent with any hypertensive disorder was associated with a lower odds of higher decreasing trajectory group. Birthing parental age, pregnancy hypertension, pre-eclampsia, gestational diabetes, preterm birth, and SGA were not associated with DBP trajectory in our population. Sensitivity models controlling for season of birth yielded similar results (Supplementary Table S7). To verify our trajectory results we also examined the relationships between BMI and elevated BP with trajectory group assignment and found that those in the higher stable group had the largest proportions of BMIs categorized as overweight and obese and of elevated BP status (Supplementary Tables S8 and S9).

**Table 5:** Unadjusted multinomial logistic regression for predictors of diastolic blood pressure (DBP) trajectory membership among NEST offspring (*n* = 1040) using higher stable^a^ as the comparison group.

	Lower increasing	Higher decreasing	*N* missing
	*n* = 166	*n* = 157	
	OR (95% CI)	OR (95% CI)	
Demographic, social, and behavioral characteristics			
Female	**1.93 (1.35–2.76)**	0.95 (0.66–1.37)	57
Birthing parent race/ethnicity			0
White^b^	Ref	Ref	
Black	**0.37 (0.25–0.53)**	**0.46 (0.31–0.68)**	
Hispanic	**0.24 (0.14–0.43)**	**0.47 (0.28–0.79)**	
Other	0.70 (0.29–1.73)	0.77 (0.30–2.00)	
Birthing parent age at delivery (years)			0
<35	Ref	Ref	
≥35	1.44 (0.95–2.18)	1.50 (0.98–2.28)	
Pre-pregnancy BMI			54
Underweight	0.91 (0.40–2.08)	0.36 (0.11–1.22)	
Normal	Ref	Ref	
Overweight	**0.60 (0.38–0.95)**	0.64 (0.40–1.01)	
Obese	**0.63 (0.42–0.94)**	**0.56 (0.37–0.87)**	
Birthing parent smoking during pregnancy	0.710.47–1.08	**0.580.37–0.92**	42
Relationship status			44
Married or living with partner	Ref	Ref	
Not married or living with partner	**0.48 (0.33–0.70)**	**0.61 (0.42–0.89)**	
Educational attainment			37
Attended college	Ref	Ref	
Did not attend college	**0.32 (0.22–0.47)**	**0.57 (0.40–0.81)**	
Birthing parent clinical characteristics			
Chronic hypertension	**0.44 (0.20–0.97)**	0.60 (0.29–1.24)	15
Pregnancy hypertension	1.15 (0.58–2.29)	0.53 (0.21–1.36)	19
Pre-eclampsia	0.67 (0.30–1.52)	0.60 (0.25–1.44)	14
Any hypertensive disorder: (chronic htn, pregnancy htn, or pre-eclampsia)	0.71 (0.43–1.15)	**0.56 (0.33–0.96)**	20
Gestational diabetes	0.78 (0.37–1.62)	1.05 (0.53–2.07)	44
Birth outcomes			
Preterm birth	1.12 (0.69–1.83)	1.20 (0.74–1.95)	12
Small for gestational age	0.93 (0.55–1.59)	0.64 (0.35–1.19)	47

*htn* hypertension.

^a^*n* = 717 participants assigned to higher stable group.

^b^Chosen reference as we consider this group “unexposed” to harmful effects of structural racism.

Bold indicates estimates with 95% confidence intervals that exclude the null.

## Discussion

In this longitudinal study, we examined predictors of elevated BP and trajectories of SBP and DBP percentiles and their associated predictors in a racially and ethnically diverse cohort of children aged 3–9. We found that infant sex assigned at birth, preterm birth, birthing parent self-reported race and ethnicity, pre-pregnancy BMI, and chronic hypertension status were associated with elevated BP among children in our study population. Additionally, we identified four distinct SBP percentile trajectories (lower stable, lower increasing, higher stable, and higher decreasing) and three distinct DBP trajectories (lower increasing, higher decreasing, and higher stable) and found that several demographic, social, lifestyle and clinical factors such as race/ethnicity, education, and the health status of gestational parents were significantly associated with offspring SBP and DBP percentile trajectory group assignment. To our knowledge, our study is the first of its kind in a U.S. cohort.

### Factors associated with elevated blood pressure

In our sample, we found that infants born preterm, those assigned male sex at birth, and those born to Black and Hispanic birthing parents had higher odds of elevated BP. Likewise, infants born to birthing parents with chronic hypertension or pre-pregnancy BMIs categorized as obese had higher odds of elevated BP. Our preterm birth finding is particularly interesting because it corroborates other studies reporting an association with elevated BP in children,^43–45^ especially among those who were extremely preterm. Additionally, several studies document an association between preterm birth and elevated BP and other cardiometabolic disorders in adulthood.^46,47^ with one study reporting a 4- and 5-fold increased risk of elevated BP in early adulthood in children in the highest SBP and DBP trajectory groups.^14^

Existing literature documents identifying or being racially designated as Black, having low SES, and experiencing adverse childhood events (ACEs; neglect, physical abuse, sexual abuse, absence of a parent, etc.) as risk factors for elevated BP in childhood and CVD morbidity later in life.^2,24,25^ We similarly identified race and SES as significant predictors of trajectory group. In fact, despite varying approaches and some differences in results across our study and previous research, there is the common thread of poorer outcomes (higher SBP trajectory group and risk for elevated BP) based on race and ethnicity, educational attainment, birthing parental health (hypertension, BMI, and diabetes) and infant health (preterm birth and rapid weight gain),^10,12,13,29,30,48^ which in many cases are intrinsically connected.

### Predictors of BP trajectories

Prior research by Yuan et al. characterized SBP trajectories from ages 3 to 8 among Singaporean children and examined associations with early life factors and childhood cardiometabolic outcomes.^30^ This group identified comparable latent trajectories (low increasing, low stable, high stable, and high decreasing) to those found in our study population. However, a smaller percentage of their sample was assigned to the “low increasing” group (18.0%) compared to our study (41.7%), and a greater proportion of their participants (47.0%) were assigned to their “high stable” group compared to our study participants (30.1%). This may be, in part, due to the larger proportion of self-reported hypertension (17%) compared to birthing parents in our study (8.0%).

The majority of previous studies used individual blood pressure measures for their trajectories and identified 3 or 4 trajectory groups,^10,29,41^ with some modeling trajectories for pre-formed groups based on race, gender, or both.^9,12,13,48,49^ The 23-year Longitudinal Georgia Stress and Heart Study was notably the first to document the association between childhood SBP trajectories and cardiovascular disease risk, particularly significant associations with intima–media thickness and left ventricular mass index.^10^

One Korean-based study examining both SBP and DBP measures in a close to age-matched cohort (3–10 years), also identified 3 DBP trajectory groups which they characterized by the pace of increase in BP and trajectory shape (low gradual or low increasing—similar to ours, moderate increase or elevated stable, and rapid increase or elevated increasing).^41^ However, this group did not report children experiencing high measures throughout the study period or measures that started high and decreased over time as we identified in our sample.

We identified several factors associated with higher stable group trajectory, including Black and Hispanic birthing parent race, pre-pregnancy BMI characterized as overweight or obese, being unmarried or living with a partner, not attending college, and gestational diabetes. Additionally, Black or Hispanic birthing parent race/ethnicity, not attending college, and chronic hypertension were associated with higher odds of offspring being in the lower increasing trajectory group. The only previous research to employ BP percentiles similarly reported high birthing parental SBP in early pregnancy and rapid postnatal weight gain (which we did not report) as prominent early life predictors of higher stable trajectory.^30^

Several previous studies using absolute values of SBP report findings similar to ours. In a Boston cohort, investigators found that higher pre-pregnancy BMI was associated with higher SBP trajectory and in adjusted models that higher SBP was associated with gestational DM.^29^ While age differences preclude direct comparisons, one study exploring trajectory assignments into adulthood also identified Black race and parental education as predictors of higher SBP trajectory group.^10^ Other studies employing absolute SBP and/ or DBP measures ranging from childhood to adulthood identified male sex, birth weight, weight gain or BMI, linear growth, family history of high BP, pregnancy hypertension, and birth order as predictors of higher SBP and DBP trajectory groups compared to the lower groups.^9,10,12,30^ Differences in results are likely due to geographical differences in study populations, and in the BP measures and statistical approaches used. Interestingly, when using percentiles, there was an additional lower stable SBP group that was not observed when using absolute measures of BP. This may be related to the incorporation of height when using percentiles, which likely makes percentiles more sensitive to outcome classification.

### Strengths and limitations

While novel, our study has several limitations that should be considered when interpreting our results. First, selection bias may have been introduced in the NEST cohort due to recruitment from prenatal care settings and the exclusion of non-English speaking individuals. Generalizability may also be limited due to the overrepresentation of highly educated individuals in our study population; however, the goal of our study was to determine trajectory patterns specific to our study participants and not to establish reference standards for the general population.

For our BP measures, it is possible that medical abstractions captured measures for children who were sick at the time of measurement, however, we did not have complete information to examine this. For this reason, we chose to utilize blood pressure measures closest to the child’s birthday to improve the chances that they are from a well visit. Additionally, there may have been some BP misclassification given the proportion of elevated BP was high in our younger aged participants (13.4% at age 3). Nonetheless, we do believe the use of SBP percentiles as opposed to individual SBP measures may have reduced the chance of outcome misclassification.^19^ Likewise, though the use of self-report of gestational parent clinical outcomes such as pre-pregnancy BMI and chronic hypertension may be subject to recall bias, we have no reason to believe this would differ by outcome and therefore any resulting bias would likely be towards the null. Additionally, we did not impute missing values as we found with the exception of preterm birth, participants included (*n* = 836) and excluded (*n* = 204) from analyses were relatively similar (Supplementary Table S10.) Still, preterm birth has been linked to higher BP,^44^ so in excluding ~20% of potentially higher risk births we may be subject to an underestimation of effects.

As with many studies, given that race and ethnicity are social constructs, our measures of race and ethnicity were limited. It is possible that the measure conflated race and ethnicity, precluding our ability to examine racial differences within the Hispanic-identifying group as well as other dimensions of race and ethnicity among all participants, such as country of origin. Despite limitations associated with our race/ethnicity measure, race was identified as one of the most important predictors of both higher stable group and elevated blood pressure, which suggests that our variable captured some of these differences at least in part.

Finally, from a statistical methods perspective, our APPs were slightly under 0.7 for the lower increasing and higher stable groups, which is below the recommended value for an ideal GBTM fit.^42^ However, the four-class linear GBTM maximized the APPs compared to other tested models including the 2, 3, and 5-class models using linear and quadratic terms, so we concluded this was the best fitting model for our data.

Despite its limitations, our study offers a number of strengths. It corroborates previous research identifying 3 to 4 trajectory groups and offers an exploration of a range of factors that may influence childhood BP in a diverse cohort. It provides a foundation for subsequent research in addition to potentially actionable insights for interventions at very young ages where they may be more feasible and have a lasting impact. While this is an epidemiologic study meant to describe a health metric in a population, it is clear that social drivers of health (SDOH) impact blood pressure trajectories in our cohort, and clinicians can bear this in mind when counseling their patients on lifestyle interventions to improve cardiovascular health. Our findings add evidence to support assessing SDOH at clinic visits to identify patients that may be at higher risk. However, future research is needed to provide specific clinical recommendations or interventions.

## Conclusion

We identified four SBP and three DBP percentile patterns in children from the ages of 3–9 that were driven by various demographic, social, behavioral, and clinical factors, some of which also contributed to elevated BP in NEST offspring. Birthing parent race and ethnicity was one of the strongest determinants of BP trajectory and elevated BP in our study, underscoring how racial disparities in CVD burden in the U.S. start extremely early in life. Our study, like many others, highlights a need for targeted interventions to address blatant disparities in health outcomes. Likewise, we identified previously documented sex disparities for elevated blood pressure status, consistent with the historically higher CVD burden among males. Considering that early cardiometabolic outcomes including elevated BP may drive adult CVD mortality and morbidity, it is increasingly important to elucidate the progression of and risk factors for these outcomes to tailor potential interventions.

## Supplementary information

4.27_Supplement_Revised 4.2025

## Data Availability

These data are available upon reasonable request.
