# Supplementary material for: Early life factors, blood pressure percentile trajectories, and elevated blood pressure in children
Source: Pediatr Res. Author manuscript; Available in PMC 2026 Jul 29. (PMC13417913; doi:10.1038/s41390-025-04307-3)
Supplement: 2 [file NIHMS2192063-supplement-2.pdf]

Supplemental Tables

Supplemental Table S1. Adjusted associations of demographic, social, behavioral, clinical, and birth outcomes with offspring elevated BP (complete cases, n=836), adjusted for season of birth.

**Characteristics**		**OR (95% CI)**
**Demographic characteristics, unadjusted**		
Female		0.64 (0.51 – 0.80)
Birthing parent race/ethnicity		
	White	Ref
	Black	1.75 (1.32 – 2.33)
	Hispanic	1.83 (1.28 – 2.60)
	Other	1.01 (0.47 – 2.15)
Birthing parent age at delivery (years)		
	<35	Ref
	>=35	1.03 (0.77 – 1.37)
**Birthing parent social and behavioral characteristics adjusted for age and race/ethnicity** [1]		
Pre-pregnancy BMI		
	Underweight	1.07 (0.59 – 1.97)
	Normal	Ref
	Overweight	1.18 (0.86 – 1.61)
	Obese	1.56 (1.18 – 2.05)
Birthing parent smoking during pregnancy		1.23 (0.94 – 1.61)
Relationship status		
	Married or cohabiting	Ref
	Not married or cohabiting	1.22 (0.94 – 1.57)
Educational attainment		
	Attended college	Ref
	Did not attend college	1.07 (0.83 – 1.38)
**Birthing parent clinical characteristics adjusted for age, race/ethnicity, and social and behavioral characteristics** [2]		
Chronic hypertension		1.48 (1.03 – 2.13)
Pregnancy hypertension		0.81 (0.49 – 1.35)
Pre-eclampsia		1.23 (0.81 – 1.87)

Any hypertensive disorder: (chronic hypertension, pregnancy hypertension, or pre-eclampsia)		1.26 (0.95 – 1.68)
Gestational DM		1.21 (0.78 – 1.86)
**Birth outcomes adjusted for age, race/ethnicity, and social, behavioral, and birthing parent clinical characteristics** [3]		
Preterm birth		1.46 (1.03 – 2.07)
Small for gestational age		0.97 (0.68 – 1.39)

[1]Adjusted for age and race/ethnicity

[2]Adjusted for age, race/ethnicity, pre-pregnancy BMI, birthing parent smoking during pregnancy, relationship status, and educational attainment

[3]Adjusted for age, race/ethnicity, pre-pregnancy BMI, birthing parent smoking during pregnancy, relationship status, educational attainment, chronic hypertension, pregnancy hypertension, pre-eclampsia, and gestational DM

Supplemental Table S2. Average posterior probability (APP) for systolic blood pressure (SBP) trajectory groups

AIC – Akaike information criterion; BIC – Bayesian Information Criterion; %class – percentage

	Group	loglikelihood	BIC n=4118	BIC n=1040	AIC	%class1	%class2	%class3	%class4	%class5
	1	-19372.03	-19384.51	-19382.45	-19375.03	100.00	NA	NA	NA	NA
	2	-19252.91	-19277.88	-19273.75	-19258.91	46.92	53.08	NA	NA	NA
	3	-19241.46	-19278.91	-19272.72	-19250.46	33.07	27.38	39.55	NA	NA
	4	-19229.02	-19278.96	-19270.7	-19241.02	19.07	35.86	27.24	17.83	NA
	5	-19228.7	-19291.12	-19280.8	-19243.7	5.26	18.56	33.60	27.55	15.03
APP						0.6964409	0.5922126	0.6769714	0.551453	

of individuals in each group; class1: low increasing, class2: low stable, class3: high stable, class4: high decreasing (class percentages based on probabilistic assignment).

Supplemental Table S3. Systolic blood pressure percentile median (IQR) by age and trajectory group

		Age						
	n	3	4	5	6	7	8	9
Trajectory								
Low stable	174	19 (12-37)	27 (14-41)	27 (15-40)	25 (17-41)	28 (18-45)	35 (20-49)	46 (24-54)
Low increasing	434	43 (23-54)	43 (31-60.5)	48 (34-70)	62 (39-78)	65 (46.5-83)	75 (54-87)	69 (55-83.5)
High stable	313	77 (61.5-89)	78 (65-88)	79 (67.5-89.5)	81 (69-90)	81 (67-93)	79 (62-92)	78 (62-91)
High decreasing	119	80 (64-88)	60 (41-81)	50.5 (33-72)	47 (27-67)	35.5 (19-63)	34 (21-52)	29 (18-46.5)

Supplemental Table S4. Unadjusted multinomial logistic regression for predictors of systolic blood pressure (SBP) trajectory membership among NEST offspring (n=1040) using lower stable* as the comparison group, adjusted for season of birth.

	**Lower increasing**	**Higher stable**	**Higher decreasing**	
	n=434	n=313	n=119	**N missing**
	**OR (95% CI)**	**OR (95% CI)**	**OR (95% CI)**	
**Demographic, social, and behavioral characteristics**				
Female	1.25 (0.87 – 1.80)	1.08 (0.74 – 1.59)	1.13 (0.70 – 1.83)	57
Birthing parent race/ethnicity				0
White**	Ref	Ref	Ref	
Black	1.59 (1.08 – 2.34)	3.70 (2.35 – 5.81)	2.06(1.19 – 3.57)	
Hispanic	3.71 (1.90 – 7.28)	10.70 (5.26 – 21.77)	5.83 (2.58–13.19)	
Other	0.68 (0.27 – 1.72)	1.53 (0.56 – 4.17)	1.43 (0.44 – 4.67)	
Birthing parent age at delivery (years)				0
<35	Ref	Ref	Ref	
≥ 35	0.84 (0.55 – 1.30)	0.68 (0.42 – 1.09)	0.88 (0.49 – 1.59)	
Pre-pregnancy BMI				54
Underweight	1.09 (0.43 – 2.72)	1.05 (0.37 – 2.97)	1.13 (0.34 – 3.82)	
Normal	Ref	Ref	Ref	
Overweight	1.04 (0.65 – 1.67)	1.73 (1.05 – 2.86)	1.34 (0.73 – 2.45)	
Obese	1.26 (0.81 – 1.94)	2.08 (1.31 – 3.32)	1.08 (0.60 – 1.94)	
Birthing parent smoking during pregnancy	0.88 (0.58 – 1.34)	1.16 (0.75 – 1.79)	0.85 (0.48 – 1.48)	42
Relationship status				44
Married or living with partner	Ref	Ref	Ref	
Not married or living with partner	1.22 (0.83 – 1.78)	1.80 (1.21 – 2.68)	1.25 (0.76 – 2.06)	
Educational attainment				37
Attended college	Ref	Ref	Ref	

Did not attend college	1.57 (1.08 – 2.29)	3.40 (2.28 – 5.07)	2.11 (1.30 – 3.42)	
**Birthing parent clinical characteristics**				
Chronic hypertension	2.39 (1.05 – 5.48)	2.29 (0.97 – 5.40)	2.08 (0.75 – 5.79)	15
Pregnancy hypertension	0.75 (0.36 – 1.56)	0.81 (0.37 – 1.75)	0.91 (0.34 – 2.41)	19
Pre-eclampsia	1.83 (0.74 – 4.53)	1.66 (0.64 – 4.29)	1.44 (0.45 – 4.59)	14
Any hypertensive disorder: (chronic htn, pregnancy htn, or pre-eclampsia)	1.41 (0.84 – 2.35)	1.36 (0.79 – 2.34)	1.64 (0.85 – 3.14)	20
Gestational diabetes	1.02 (0.43 – 2.38)	2.25 (0.99 – 5.08)	2.94 (1.16 – 7.43)	44
**Birth outcomes**				
Preterm birth	1.26 (0.71 – 2.21)	1.67 (0.94 – 2.97)	1.20 (0.58 – 2.51)	12
Small for gestational age	1.20 (0.67 – 2.16)	1.31 (0.72 – 2.41)	1.20 (0.56 – 2.56)	47

Note: htn – hypertension

*n=174 participants assigned to lower stable group

**Chosen reference as we consider this group "unexposed" to harmful effects of structural racism.

Supplemental Table S5. Average posterior probability (APP) for diastolic blood pressure (DBP) trajectory groups

	Group	loglikelihood	BIC n=4118	BIC n=1040	AIC	%class 1	%class 2	%class3	%class4	%class5
	1	-18727.63	-18740.12	-18738.05	-18730.63	100.00	NA	NA	NA	NA
	2	-18631.79	-18656.76	-18652.63	-18637.79	16.01	83.99	NA	NA	NA
	3	-18621.78	-18659.23	-18653.04	-18630.27	15.62	30.14	54.25	NA	NA
	4	-18618.27	-18668.21	-18659.95	-18630.27	4.24	15.16	31.11	49.27	NA
	5	-18727.63	-18740.12	-18738.05	-18730.63	100.00	NA	NA	NA	NA
APP						0.7191331	0.6150066	0.691592		

AIC – Akaike information criterion; BIC – Bayesian Information Criterion; %class – percentage of individuals in each group; class1: lower increasing, class2: higher decreasing, class3: higher stable (class percentages based on probabilistic assignment).

Supplemental Table S6. Diastolic blood pressure percentile median (IQR) by age and trajectory group

		Age						
	n	3	4	5	6	7	8	9
Trajectory								
Lower increasing	166	32.5 (20-50)	40 (24-54)	42 (28-63)	52 (31-63)	52 (38.5-63)	57 (47-69)	61 (43-69)
Higher decreasing	157	81 (68.5.5-90)	77 (59-86)	59 (46-75)	45 (30-64)	48 (27.5-55)	45 (29-56)	41.5 (26-51)
Higher stable	717	82 (66-91)	81 (63-91)	75 (59-87)	72 (58-87)	75 (59-89)	70 (55-87)	70 (57-82)

Supplemental Table S7. Unadjusted multinomial logistic regression for predictors of diastolic blood pressure (DBP) trajectory membership among NEST offspring (n=1040) using higher stable* as the comparison group, adjusted for season of birth

	**Lower increasing**	**Higher decreasing**	
	n=166	n=157	**N missing**
	**OR (95% CI)**	**OR (95% CI)**	
**Demographic, social, and behavioral characteristics**			
Female	**1.90 (1.33 – 2.71)**	0.96 (0.67 – 1.38)	57
Birthing parent race/ethnicity			0
White**	Ref	Ref	
Black	**0.37 (0.25 – 0.54)**	**0.46 (0.31 – 0.69)**	
Hispanic	**0.24 (0.14 – 0.43)**	**0.46 (0.28 – 0.78)**	
Other	0.73 (0.29 – 1.80)	0.77 (0.29 – 2.01)	
Birthing parent age at delivery (years)			0
<35	Ref	Ref	
≥ 35	1.42 (0.94 – 2.16)	1.50 (0.98 – 2.29)	
Pre-pregnancy BMI			54
Underweight	0.92 (0.40 – 2.12)	0.35 (0.10 – 1.19)	
Normal	Ref	Ref	
Overweight	**0.61 (0.38 – 0.96)**	**0.63 (0.40 – 1.00)**	
Obese	**0.62 (0.42 – 0.94)**	**0.56 (0.37 – 0.86)**	
Birthing parent smoking during pregnancy	0.72 (0.47 – 1.09)	**0.59 (0.38 – 0.93)**	42
Relationship status			44
Married or living with partner	Ref	Ref	
Not married or living with partner	**0.47 (0.32 – 0.69)**	**0.62 (0.43 – 0.90)**	
Educational attainment			37
Attended college	Ref	Ref	
Did not attend college	**0.32 (0.22 – 0.47)**	**0.57 (0.40 – 0.82)**	
**Birthing parent clinical characteristics**			
Chronic hypertension	**0.43 (0.19 – 0.96)**	0.60 (0.29 – 1.22)	15
Pregnancy hypertension	1.11 (0.56 – 2.22)	0.52 (0.20 – 1.33)	19
Pre-eclampsia	0.68 (0.30 – 1.54)	0.60 (0.25 – 1.45)	14
Any hypertensive disorder: (chronic htn, pregnancy htn, or pre-eclampsia)	0.70 (0.43 – 1.15)	**0.55 (0.32 – 0.95)**	20
Gestational diabetes	0.73 (0.35 – 1.53)	1.05 (0.53 – 2.09)	44

**Birth outcomes**			
Preterm birth	1.15 (0.71 – 1.88)	1.20 (0.74 – 1.96)	12
Small for gestational age	0.94 (0.55 – 1.61)	0.63 (0.34 – 1.17)	47

Note: htn – hypertension
[*]n=174 participants assigned to lower stable group
[**]Chosen reference as we consider this group "unexposed" to harmful effects of structural racism.

Supplemental Table S8. Number and proportion of offspring with BMIs categorized as overweight/ obese by trajectory group and age in The Newborn Epigenetic STudy (NEST) Children n=1040

	**Age**						
	3	4	5	6	7	8	9
**Trajectory**	n (%)	n (%)	n (%)	n (%)	n (%)	n (%)	n (%)
Lower stable	27 (18.5)	29 (20.0)	32 (21.2)	29 (25.0)	30 (28.3)	25 (28.4)	23 (30.3)
Lower increasing	84 (22.8)	110 (29.6)	104 (28.0)	88 (31.9)	91 (40.3)	80 (38.5)	67 (39.9)
Higher stable	98 (38.3)	116 (44.4)	127 (47.2)	105 (52.5)	91 (52.0)	83 (55.7)	79 (63.2)
Higher decreasing	25 (23.8)	26 (24.5)	31 (28.2)	24 (24.7)	26 (27.7)	30 (32.6)	27 (36.5)
Missing	n=165	n=156	n=139	n=351	n=439	n=503	n=597

Supplemental Table S9. Number and proportion of offspring with elevated BP by trajectory group and age in The Newborn Epigenetic STudy (NEST) Children (n=836)

	**Age**						
	3	4	5	6	7	8	9
**Trajectory**	n (%)	n (%)	n (%)	n (%)	n (%)	n (%)	n (%)
Lower stable	1 (1.0)	10 (8.5)	2 (1.6)	0 (0)	0 (0)	0 (0)	0 (0)
Lower increasing	12 (4.8)	23 (7.7)	17 (5.6)	11 (5.3)	19 (13.4)	9 (9.9)	3 (5.5)
Higher stable	41 (27.2)	40 (20.8)	34 (16.8)	33 (24.4)	24 (24.2)	17 (27.0)	6 (16.7)
Higher decreasing	20 (26.0)	12 (13.6)	4 (4.5)	0 (0)	3 (4.4)	0 (0)	0 (0)
No. with elevated BP	n=74	n=85	n=57	n=44	n=46	n=26	n=9
**Total**	n=580	n=696	n=717	n=506	n=389	n=242	n=137

Supplement Table S10. Comparisons of covariates across complete cases and participants with missing covariate data

**Characteristics**	**Complete Cases (n=836)**	**Participants with Missing Covariate Data (n=204)**	**Missing n (%)**
**Birthing parent age at delivery**	28.4 (6.3)	28.4 (6.0)	0
**Birthing parent race/ethnicity**			
Black	415 (54.9)	112 (49.6)	0
White	239 (28.6)	49 (24.0)	
Hispanic	157 (18.8)	34 (16.7)	
Other	25 (3.0)	9 (4.4)	
**Pre-pregnancy BMI**			54 (5.2)
Underweight	34 (4.1)	5 (3.3)	
Normal	323 (38.6)	51 (34.0)	
Obese	280 (33.5)	53 (35.3)	
Overweight	199 (23.8)	41 (27.3)	
**Birthing parent smoking during pregnancy**			42 (4.0)
No	645 (77.2)	106 (65.4)	
Yes	191 (22.8)	56 (34.6)	
**Attended college**			37 (3.6)
No	405 (48.4)	82 (49.1)	
Yes	431 (51.6)	85 (50.9)	
**Married or cohabitating**			44 (4.2)
No	325 (38.9)	62 (38.8)	
Yes	511 (61.1)	98 (61.2)	
**Pre-eclampsia**			14 (1.35)
No	785 (93.9)	184 (96.8)	
Yes	51 (6.1)	6 (3.2)	
**Gestational diabetes**			44 (4.2)
No	785 (93.9)	143 (89.4)	
Yes	51 (6.1)	17 (10.6)	
**Chronic hypertension**			15 (1.4)
No	766 (91.6)	177 (93.7)	
Yes	70 (8.4)	12 (6.4)	
**Pregnancy hypertension**			19 (1.8)
No	787 (94.1)	176 (95.1)	

Yes	49 (5.9)	9 (4.9)	
**Any hypertensive disorder: (chronic hypertension, pregnancy hypertension, or pre-eclampsia)**			20 (1.9)
No	691 (82.7)	162 (88.0)	
Yes	145 (17.3)	22 (12.0)	
**Sex of Baby**			57 (5.5)
Male	432 (51.7)	82 (55.8)	
Female	404 (48.3)	65 (44.2)	
**Preterm birth**			12 (1.2)
No	755 (90.3)	132 (68.8)	
Yes	81 (9.7)	60 (31.3)	
**Small for gestational age**			47 (4.5)
No	737 (88.2)	135 (86.0)	
Yes	99 (11.8)	22 (14.0)	
